# L‐carnitine for valproic acid‐induced toxicity

**DOI:** 10.1111/bcp.16233

**Published:** 2024-09-11

**Authors:** Tomasz Gziut, Ruben Thanacoody

**Affiliations:** ^1^ National Poisons Information Service (Newcastle unit) Newcastle‐upon‐Tyne Hospitals NHS Foundation Trust UK; ^2^ Translational and Clinical Research Institute Newcastle University Newcastle‐upon‐Tyne UK

**Keywords:** hyperammonaemia, L‐carnitine, overdose, valproic acid

## Abstract

**Aims:**

Review the effectiveness and dosing of L‐carnitine for valproic‐acid induced toxicity.

**Methods:**

A literature review of the pharmacokinetics and clinical use of L‐carnitine was performed.

**Results:**

Valproic acid is a fatty acid used for numerous therapeutic indications ranging from epilepsy to bipolar disorder. The metabolism of valproic acid produces both therapeutic and toxic metabolites. Whilst it has a good safety profile, adverse effects of valproic acid in chronic use include hepatotoxicity ranging from transient elevation of liver enzymes to fulminant liver failure and hyperammonaemia with resultant encephalopathy. L‐carnitine is an essential cofactor for mitochondrial fatty acid metabolism, which is an important source of energy in cardiac and skeletal muscle. Physiological concentrations of L‐carnitine are maintained in man by exogenous dietary intake and endogenous synthesis. Following exogenous oral administration of L‐carnitine, the bioavailability ranges from 14% to 18%. After bolus intravenous administration of L‐carnitine in doses ranging from 20 to 100 mg/kg, the volume of distribution is 0.2–0.3 L/kg, and the fraction excreted unchanged in urine is 0.73–0.95, suggesting that renal clearance of L‐carnitine is dose dependent due to saturable renal reabsorption at supraphysiological concentrations.

**Conclusions:**

There is evidence supporting the use of L‐carnitine in treating hyperammonaemia and hepatotoxicity following chronic therapeutic use and after acute overdose of valproic acid, but the optimal dose and route of administration is unknown. Based on the pharmacokinetics of L‐carnitine, we advocate the administration of L‐carnitine for valproic‐acid induced hyperammonaemia or hepatotoxicity as an intravenous loading dose of 5 mg/kg followed by a continuous intravenous infusion instead of the oral or intravenous boluses that are currently advocated.

## 1 INTRODUCTION

Valproic acid (VPA) is a commonly used antiepileptic drug licensed for the treatment of partial and generalized seizures, as well as bipolar affective disorder and migraine prophylaxis. Toxicity can occur in therapeutic use or after overdose. L‐carnitine has been suggested as an antidote for the management of VPA ‐induced toxicity.

In the UK, L‐carnitine is licensed for the treatment of primary carnitine deficiency due to inborn errors of metabolism and for secondary carnitine deficiency in haemodialysis patients. In the paediatric population, it is also licensed for use in organic acidaemias. Dosing recommendations are shown in Table 1.

**TABLE 1:** Dosing recommendations for L‐carnitine.

Indication	Recommendations
*Primary carnitine deficiency*	**Adult (PO)** Up to 200 mg/kg daily in 2–4 divided doses; maximum 3 g/day. **Adult (IV)** Up to 100 mg/kg daily in 2–4 divided doses, to be administered over 2–3 min.
*Secondary carnitine deficiency in haemodialysis patients*	**Adult** 20 mg/kg, to be administered over 2–3 min, after each dialysis session, dosage adjusted according to plasma‐carnitine concentration, then (by mouth) maintenance 1 g daily, administered if benefit is gained from first intravenous course.
*Inborn errors of metabolism*	**Neonate (PO)** Up to 200 mg/kg daily in 2–4 divided doses. **Child (PO)** Up to 200 mg/kg daily in 2–4 divided doses; maximum 3 g/day. **Neonate (initially by IV infusion)** Initially 100 mg/kg, to be administered over 30 min, followed by (by continuous intravenous infusion) 4 mg/kg/h. **Child (initially by IV infusion)** Initially 100 mg/kg, to be administered over 30 min, followed by (by continuous intravenous infusion) 4 mg/kg/h. **Neonate (slow IV injection)** Up to 100 mg/kg daily in 2–4 divided doses, to be administered over 2–3 min. **Child (slow IV injection)** Up to 100 mg/kg daily in 2–4 divided doses, to be administered over 2–3 min.
*Secondary carnitine deficiency in haemodialysis patients*	**Child (slow IV Injection)** 20 mg/kg, to be administered over 2–3 min, after each dialysis session, dosage adjusted according to plasma‐carnitine concentration, then (by mouth) maintenance 1 g daily, administered if benefit is gained from first intravenous course.
*Organic acidaemias*	**Neonate (PO)** Up to 200 mg/kg daily in 2–4 divided doses. **Child (PO)** Up to 200 mg/kg daily in 2–4 divided doses; maximum 3 g/day. **Neonate (IV infusion)** Initially 100 mg/kg, to be administered over 30 min, followed by (by continuous intravenous infusion) 4 mg/kg/h. **Child (IV Infusion)** Initially 100 mg/kg, to be administered over 30 min, followed by (by continuous intravenous infusion) 4 mg/kg/h. **Neonate (slow IV injection)** Up to 100 mg/kg daily in 2–4 divided doses, to be administered over 2–3 min. **Child (slow IV injection)** Up to 100 mg/kg daily in 2–4 divided doses, to be administered over 2–3 min.
Guideline dosing recommendations for L‐carnitine	
**Consensus guidelines for management of hyperammonaemia in paediatric patients receiving continuous renal replacement therapy** ^1^	**IV**: L‐carnitine: 50 mg/kg loading dose given over 90 min, then 100–300 mg/kg daily (not needed in patients with urea cycle disorders but needed in patients with organic acidaemias).
**L‐carnitine supplementation in childhood epilepsy: current perspectives (1998)** ^2^	**PO**: Daily dose should be 100 mg/kg/day, or 2 g/day, whichever is less in 3 or 4 divided doses. **IV**: for *metabolic rescue* of acutely ill patients, L‐ carnitine in higher doses of 150–500 mg/kg/day are recommended.

*Note*: British National Formulary dosing recommendations.^3^, ^4^

Abbreviations: IV, intravenous; PO, oral.

L‐carnitine has also been used off‐label in the management of acute or chronic overdose of sodium valproate in the presence of hepatotoxicity, hyperammonaemic encephalopathy, hepatotoxicity or coma. The use of L‐carnitine in patients with overdose of sodium valproate is based on the theoretical mechanism of action and case reports.

This review covers the pathogenesis of VPA‐induced toxicity, the mechanistic rationale for use of L‐carnitine as an antidote and the implications of the pharmacokinetics of exogenously administered L‐carnitine for therapeutic dosing.

## 2 L‐CARNITINE

L‐carnitine (3‐hydroxy‐4‐N‐trimethylammonium butyrate) is a water‐soluble amino acid with a molecular weight of 161 Da. The main physiological function of L‐carnitine is to facilitate transport of fatty acids into the mitochondria via the carnitine shuttle where β‐oxidation can occur. L‐carnitine is also vital for the removal of xenobiotics, stabilization of cell membranes, removal of toxic metabolites and maintaining the balance of the coenzyme A (CoA) pool in mitochondria.^5^

### 2.1 L‐carnitine and fatty acid metabolism

Fatty acids enter the cytosol of cells via fatty acid transport proteins 1–6, as well as caveolins and plasma membrane fatty acid binding proteins.^6^, ^7^ Fatty acid translocase also facilitates fatty acid movement, but has many other functions ranging from taste perception of fats to the regulation of insulin release.^8^

**FIGURE 1:**
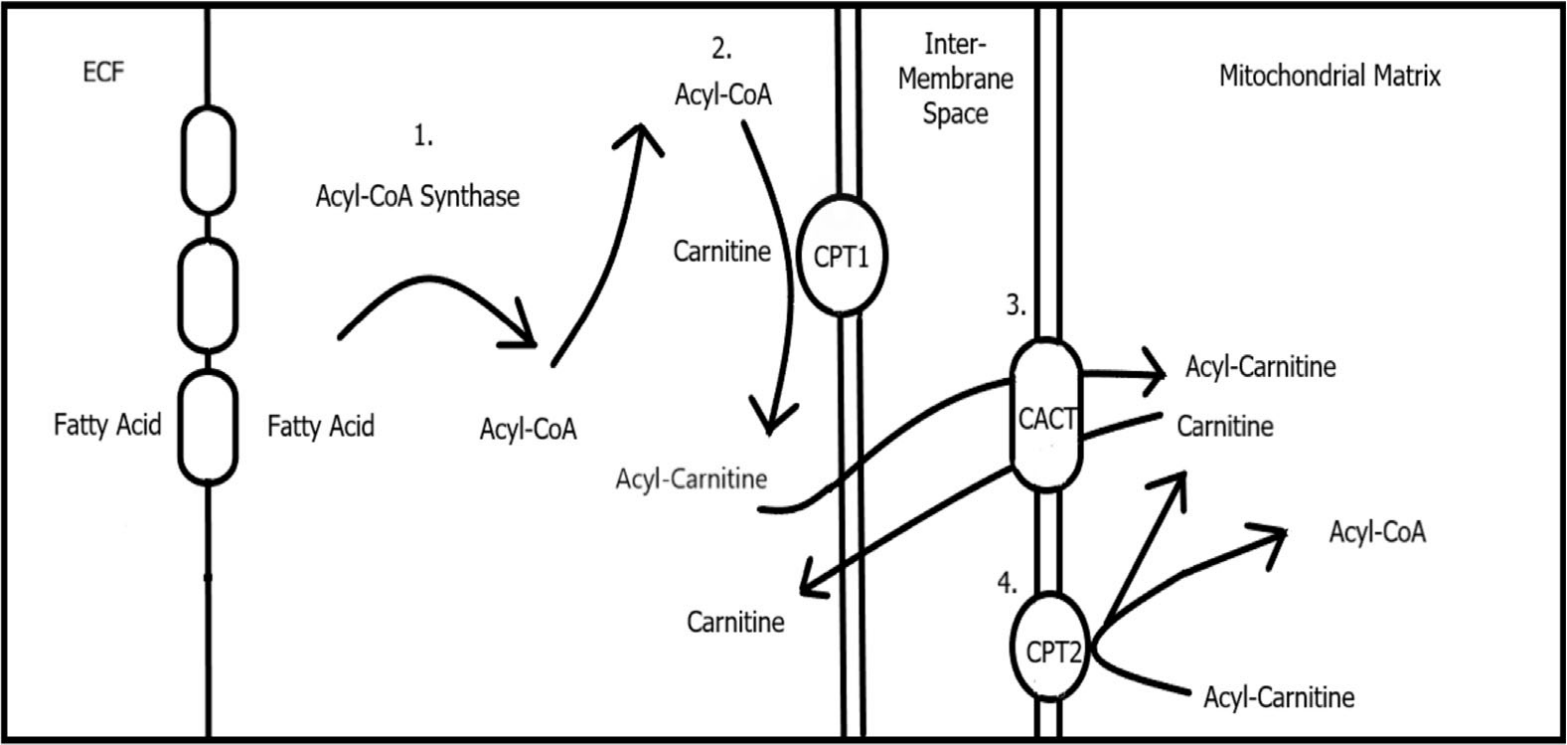
Fatty‐acid mitochondrial β‐oxidation. ECF, extracellular fluid; CPT1, carnitine palmitoyltransferase 1; CACT, carnitine‐acylcarnitine translocase; CPT2, carnitine palmitoyltransferase 2. Steps: 1. Fatty acids conversion to acyl‐CoA by acyl‐CoA synthase. 2. Acyl‐CoA and carnitine conversion to acylcarnitine by CPT1. 3. Acylcarnitine enters the mitochondrial intermembrane space and transported into mitochondrial matrix by CACT. 4. Acylcarnitine converted back to acyl‐CoA and carnitine by CPT2.

The initial step of fatty acid transport into the mitochondria requires the conversion of fatty acids to acyl‐CoA by acyl‐CoA synthase. The movement of acyl‐CoA from the cytosol, across the outer mitochondrial membrane, the intermembrane space and the inner mitochondrial membrane and into the mitochondrial matrix is a multi‐step process facilitated by L‐carnitine and is known as the carnitine shuttle (Figure 1).

Transport of acyl‐CoA from the cytosol to the mitochondrial matrix starts with L‐carnitine accepting an acyl group from acyl‐CoA via the enzyme carnitine acyltransferase‐1 (carnitine palmytoyltransferase 1) to form acyl‐carnitine. Acyl‐carnitine crosses the outer mitochondrial membrane by passive diffusion and enters the mitochondrial intermembrane space. Acylcarnitine is then transported via the membrane‐bound carnitine–acylcarnitine translocase (CACT) into the mitochondrial matrix. Inside the mitochondrial matrix, acylcarnitine is converted back to acyl‐CoA and L‐carnitine by carnitine acyltransferase‐2 (carnitine palmitoyltransferase 2). Acyl‐CoA undergoes β‐oxidation within the mitochondrial matrix, whilst the carnitine is returned to the cytosol via the CACT cotransporter.^6^

### 2.2 Endogenous synthesis and distribution of L‐carnitine

The majority of dietary L‐carnitine is obtained from meat and dairy products. Fruit and vegetables contain negligible amounts of L‐carnitine and dietary intake can vary between vegetarians and nonvegetarians. In strict vegetarians, >90% of L‐carnitine is provided via endogenous synthesis. Renal reabsorption of L‐carnitine is also very efficient (90–99% of the filtered load) and is crucial for maintaining serum L‐carnitine concentrations. The interplay between dietary intake, endogenous synthesis and renal reabsorption allow for tightly controlled concentrations of L‐carnitine in humans.^9^

The plasma concentrations of L‐carnitine and acetyl‐L‐carnitine in healthy adults are generally 40–50 μmol/L and 3–6 μmol/L respectively. The total L‐carnitine concentration (including free L‐carnitine and carnitine esters) is about 50–60 μmol/L. Tissue concentrations of L‐carnitine are significantly higher, ranging from 2000 to 4000 μmol/L in skeletal muscle, 500–1000 μmol/L in liver and 300–600 μmol/L in kidney. The total carnitine pool in the average healthy male is about 20 g, of which >98% can be found in skeletal muscle, 1% in the liver and approximately 0.4% in plasma. The high tissue to plasma ratio of L‐carnitine is partly maintained by the carnitine organic cation transporter, which is widely expressed in human tissue and is also responsible for the tubular reabsorption of L‐carnitine.^10^ The published Km values for uptake into different tissues vary significantly, ranging from 20–100 μmol/L for skeletal and cardiac muscle, 0.1–0.5 mmol/L for kidney, and 1–5 mmol/L for liver and brain.^10^

Endogenous multistep synthesis of L‐carnitine from lysine and methionine takes place in the kidney, brain and liver but not in cardiac and skeletal tissue; they depend on carrier‐mediated uptake of L‐carnitine from blood. As L‐carnitine is not plasma protein bound, it undergoes extensive glomerular filtration and blood levels of L‐carnitine are maintained by extensive tubular reabsorption such that >98% of L‐carnitine is reabsorbed under normal physiological conditions. Renal excretion of L‐carnitine in vegan or vegetarian individuals is greatly decreased, which helps to maintain homeostasis.^10^ The threshold concentration for tubular reabsorption of L‐carnitine in healthy adults is 40–60 μmol/L, similar to the endogenous plasma L‐carnitine concentration.

The distribution of carnitine stores needs to be considered for the interpretation of the clinical significance of serum carnitine levels in disease states, especially when the steady state is disturbed acutely or after supplementation of L‐carnitine orally or intravenously (IV).

### 2.3 Pharmacokinetics of exogenous L‐carnitine

L‐carnitine is available in capsules or as an oral solution. It can also be used IV (intravenously). It is well tolerated with nausea and vomiting being the most commonly reported side‐effects. Care needs to be taken to use only L‐carnitine, given that its D‐ isomer depletes the L‐ isomer and can cause toxicity.

Following oral administration, uptake of L‐carnitine into the small intestinal epithelium occurs partly by carrier‐mediated transport and partly by passive diffusion. Whilst L‐carnitine is absorbed efficiently at physiological levels (with only 4% of tracer labelled L‐carnitine detected in the faeces of healthy adults eating a normal diet), pharmacokinetic studies of larger doses (2–6 g) of L‐carnitine in healthy volunteers consistently show poor oral bioavailability of 5–18%. The hepatic extraction ratio in humans is low, suggesting that the low oral bioavailability results from the high polarity of L‐carnitine limiting passive diffusion, the limited capacity of intestinal transporters and acetylation in the intestinal epithelium.^10^

The bioavailability of L‐carnitine is dose‐dependent and reduces as transporters become saturated. The reported absolute bioavailability of L‐carnitine following single oral doses of 100 mg/kg solution, 2 g tablets and 6 g tablets were 18, 16 and 14%, respectively.^11^, ^12^, ^13^ In the study by Harper *et al*., the bioavailability following 6 g dose was 5% but dissolution may have been affected by the large number of tablets (18) in a small volume (400 mL) of water.^13^

Following repeated administration of 2 g of L‐carnitine orally every 12 h for 4 days in 3 different formulations (chewable tablet, oral solution and tablet), the time to maximum concentration was 3.1–3.4 h, with a peak plasma concentration of 53.8–54.2 μmol/L and trough plasma concentration of 53.8–54.5 μmol/L.^14^ Another study of oral L‐carnitine in doses of 0.5 g, 1 g and 2 g 3 times daily for 7 days showed no significant increase in serum L‐carnitine levels above the 0.5 g dose.^15^ At higher doses, L‐carnitine is metabolized to trimethylamine and γ‐butyrobetaine. Trimethylamine is absorbed from the gut and metabolized to trimethylamine‐N‐oxide in the liver whilst γ‐butyrobetaine is excreted faecally. A small amount may be absorbed and converted to L‐carnitine in the liver.^2^

After IV administration of L‐carnitine in doses ranging from 20 to 100 mg/kg, the initial volume of distribution was 0.2–0.3 L/kg, the terminal half‐life ranged from 4 to 23 h and plasma concentrations of L‐carnitine returned to baseline within 12–24 h. The fraction of L‐carnitine excreted unchanged in urine was 0.73–0.95, suggesting that although renal reabsorption of L‐carnitine is very efficient at physiological concentrations, renal transporters become saturated at the high concentrations achieved with IV dosing resulting in extensive renal excretion. Under physiological baseline conditions, the renal clearance of L‐carnitine is 1–3 mL/minute but increases to 78 mL/min following a 2‐g IV bolus dose and 100 mL/min after a 6‐g IV dose. Administration of L‐carnitine during haemodialysis showed that the extraction ratio of L‐carnitine is 74%.^10^

The distribution of exogenous L‐carnitine was characterized in a study using an IV tracer dose of L‐[methyl‐ 3H] carnitine in 6 healthy volunteers with blood sampling for 28 days. Pharmacokinetic modelling suggested a 3‐compartmental distribution with mean residence times of 1 h (central compartment), 12 h (fast equilibrating compartment such as liver and kidney) and 191 h (slow equilibrating compartment such as skeletal and cardiac muscle), suggesting that transfer of L‐carnitine from plasma to the main carnitine pool is a slow process taking days to weeks.^16^

## 3 VALPROIC ACID

VPA is a branched chain carboxylic acid initially discovered in 1882 by Beverly Burton but did not enter the UK market until 1973.^17^ It is available in various formulations including IV injection, standard release tablets, prolonged release tablets, capsules and granules, and as a liquid formulation.

### 3.1 Pharmacokinetics of VPA

VPA is well absorbed with an oral bioavailability >90%. VPA is >90% bound to plasma protein and has a small volume of distribution. Its elimination half‐life is between 10 and 16 h and steady state concentrations are usually achieved within 2–3 days.^18^

At therapeutic levels, VPA is metabolized in the liver through 3 pathways: glucuronide conjugation; β‐oxidation in the mitochondrial matrix; and ω‐oxidation (CYP450 metabolism) in the cytosol. The majority of VPA metabolism is through the first 2 pathways, with approximately 10% of VPA metabolized via the CYP450 pathway.^17^ The metabolism of VPA via these 3 metabolic pathways produces both therapeutic and toxic metabolites, with over 50 different metabolites discovered so far.^19^ Glucuronide conjugation of VPA leads to the formation of valproate acid glucuronide and 2‐n‐propyl‐2‐pentenoic acid, both of which are therapeutic metabolites.

Beta‐oxidation of fatty acids in the mitochondria eventually leads to the formation of 2‐carbon units which become acetyl‐CoA after binding with coenzyme A. Acetyl‐CoA is transformed into citrate, which enters the Krebs cycle; hence, fatty acid β‐oxidation is the main energy source for cells. The presence of L‐carnitine is necessary for fatty acids to enter the mitochondria and, as such, its presence and concentration is a rate‐limiting step in cellular fatty acid metabolism.

Valproic acid, a branched chain carboxylic acid, binds to coenzyme A in the cytosol to form valproyl‐CoA, which appears to be a therapeutic metabolite but may lead to mitochondrial DNA depletion in susceptible individuals and is postulated to cause hepatotoxicity. Valproyl‐CoA requires L‐carnitine for transfer via the carnitine shuttle to the mitochondrial matrix where β‐oxidation occurs. 2‐en‐VPA is also produced during β‐oxidation and is neurotoxic and can cause symptoms such as diplopia, ataxia, seizures and cognitive disorders.^20^

The toxic metabolites 3‐hydroxy‐VPA, 4‐hydroxy‐VPA and 5‐hydroxy‐VPA and 4‐en‐VPA are all by‐products of CYP450 metabolism and are hepatotoxic. 2‐N‐propyl‐4‐oxopentanoic acid has been shown to cause neural tube defects, microvesicular steatosis and reduces the excretion of ammonia via carbamoyl phosphate synthase. 2‐Propyl‐2,4‐pentadienoic acid is formed during CYP450 metabolism and has been shown to inhibit β‐oxidation leading to reduced levels of acetyl‐CoA and glutamate. Without glutamate and acetyl‐CoA, synthesis of N‐acetylglucosamine 6‐phosphate deacetylase is decreased, which impairs the activation of carbamoyl phosphate synthase 1 (CPS1) leading to hyperammonaemia.^20^

VPA metabolism is shifted towards ω‐oxidation when L‐carnitine is depleted and/or when VPA concentrations exceed therapeutic levels such as in acute overdose or high dose chronic use. This in turn can produce toxic metabolites responsible for inhibiting CPS1. CPS1 is needed to catalyse the binding of ammonia to bicarbonate in the mitochondria. Without this step, ammonia cannot enter the urea cycle and be excreted in urine. CPS1 inhibition thus leads to increased levels of serum ammonia.^21^

### 3.2 Mechanism of action of VPA

VPA decreases the degradation of γ‐aminobutyric acid (GABA), increases GABA production and acts directly on GABA‐ A and GABA‐B receptors to increase the inhibitory function of GABA. These mechanisms underpin its action as an antiepileptic drug.^22^, ^23^ VPA is also thought to decrease neuronal firing by inhibiting sodium and calcium channels. It has also been found to increase levels of extracellular serotonin and dopamine, which may explain its activity for bipolar disorder.^17^

### 3.3 Valproic acid‐induced toxicity

VPA is well tolerated in the majority of patients but has well‐documented undesirable effects. Gastrointestinal side‐effects such as nausea, vomiting and (rarely) pancreatitis have been reported. Neurological side‐effects such as tremor, headache and extrapyramidal symptoms are possible. Haematological side‐effects (anaemia and thrombocytopenia) and endocrine system disruption (syndrome of inappropriate antidiuretic hormone secretion and dysmenorrhoea) have also been documented.^24^ Hyperammonaemia without clinically significant liver injury has been very well studied and is most likely linked to mitochondrial dysfunction.

VPA induced hepatotoxicity is a well‐documented undesirable effect and can present clinically in different forms such as an asymptomatic and reversible elevation of aminotransferases (alanine transaminase or aspartate aminotransferase), hyperammonaemia with minimal or no liver injury and acute hepatocellular injury with jaundice which can progress to fulminant liver failure. A Reye‐like syndrome in children treated with VPA is also a recognized phenomenon.

Transient asymptomatic elevations in liver enzymes appear to be fairly common and usually resolve after discontinuation of VPA. Whilst 5–10% of patients taking VPA may develop a transient alanine transaminase rise, numerous cases of acute liver injury presenting with jaundice and progression to fatal liver failure have been reported, with most cases occurring within the first 6 months of treatment. The histopathological findings are microvesicular steatosis, inflammation and cholestasis.

The pathogenesis of VPA‐induced hepatotoxicity is multi‐factorial. Patients with inborn errors of metabolism or genetic variants of enzymes involved in VPA metabolism appear to be at risk of developing hepatotoxicity. There is good evidence to suggest CYP450 polymorphisms and polypharmacy with other antiepileptic medications and HIV medications can predispose to development of liver toxicity. Inhibition of β‐oxidation, after acute VPA overdose or due to L‐carnitine deficiency, has also been studied as a predisposing factor.^25^

### 3.4 Valproic acid and hyperammonaemia

Although ammonia is produced in all cells, the majority is produced in the gastrointestinal tract (via bacterial breakdown of circulating urea, breakdown of circulating glutamine and deamination of ingested proteins), skeletal muscle (after physical activity or seizures and due to amino acid metabolism) and the kidney (via deamidation of adenosine monophosphate). Ammonia is able to cross the blood–brain barrier and is neurotoxic with cerebral concentrations usually twice that of serum concentrations. Cerebral oedema is postulated to occur due to astrocyte swelling secondary to accumulation of glutamine in glial cells.

**FIGURE 2:**
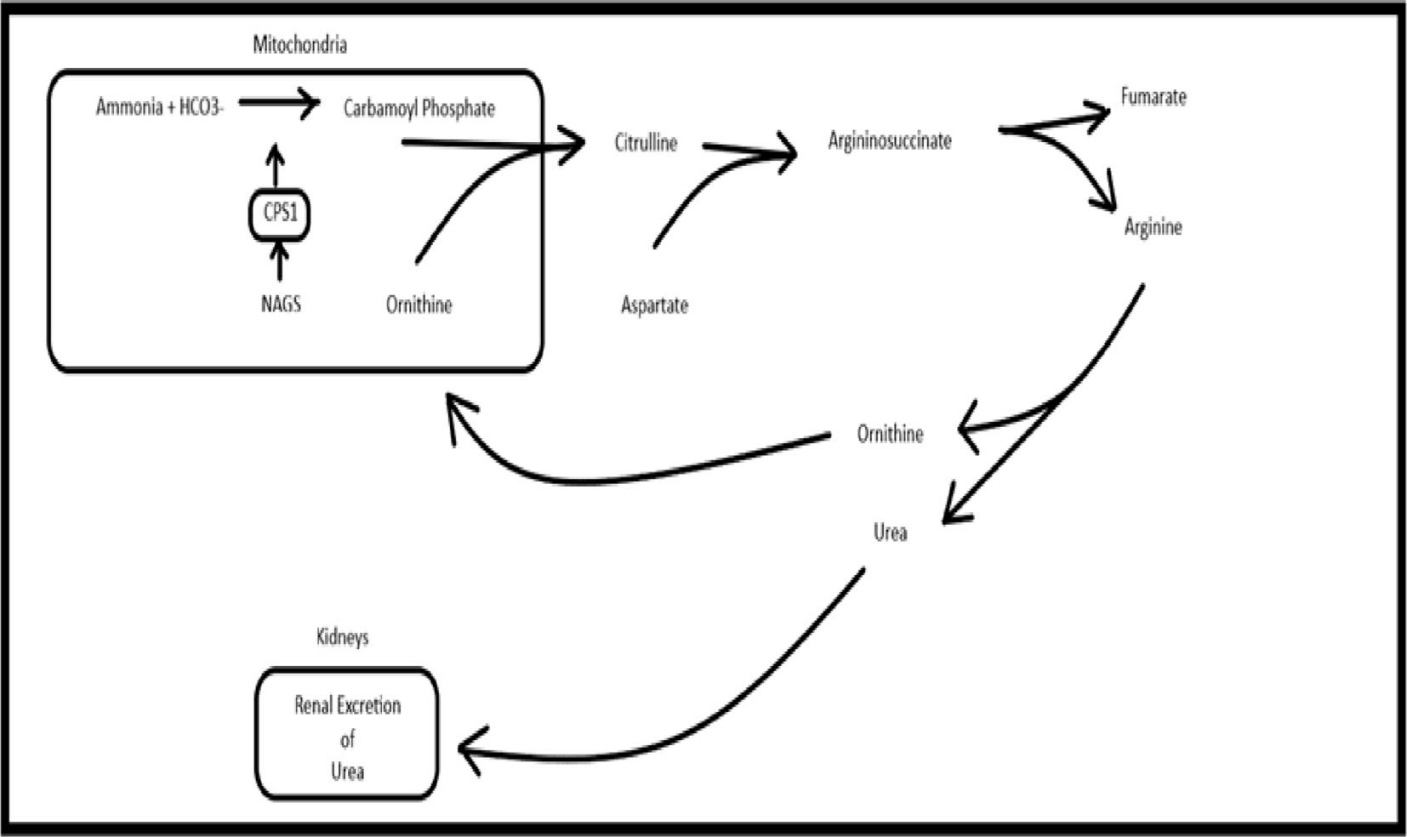
Urea cycle. CPS1, carbamoyl phosphate synthase 1; NAGS, N‐acetyl glutamate synthase. Steps: 1. NAGS activates CPS1. 2. CPS1 catalyses the conversion of ammonia and bicarbonate to carbamoyl phosphate. 3. Ornithine transcarbamoylase catalyses the conversion of ornithine and carbamoyl phosphate to citrulline. 4. Citrulline combines with aspartate to form arginosuccinate. 5. Argininosuccinate lyase breaks down arginosuccinate to form arginine and fumarate. 6. Arginine is broken down by arginase to form urea and ornithine. 7. Ornithine re‐enters the mitochondrial matrix. Urea is excreted via the kidneys.

Ammonia is converted to urea in the liver via the urea cycle and is subsequently excreted by the kidneys.^26^ The first ATP‐dependent step of the urea cycle occurs in the mitochondria when ammonia combines with bicarbonate to form carbamoyl phosphate, catalysed by CPS1. CPS1 requires N‐acetyl‐glutamate synthesized from glutamate and acetyl‐CoA via N‐acetyl‐glutamate synthase (NAGS) as an obligate activator. Carbamoyl phosphate combines with ornithine via ornithine transcarbamylase to form citrulline which then exits the mitochondria. In the cytosol, citrulline and aspartate form argininosuccinate which is converted to arginine and fumarate via argininosuccinate lyase. Arginine is hydrolysed via arginase to form urea and ornithine. Ornithine re‐enters the mitochondria to bind to carbamoyl phosphate and the cycle is repeated.^27^, ^28^ (Figure 2).

VPA is thought to cause hyperammonaemia by 3 proposed mechanisms: (i) inhibition of CPS1 by VPA or 1 of its metabolites; (ii) interference with NAGS, which activates CPS1; (iii) inhibition of fatty acid oxidation either through the depletion of L‐carnitine or depletion of acetyl‐CoA (a substrate of NAGS). Increased levels of valproyl‐CoA and propionyl‐CoA inhibit the activity of NAGS and reduced NAGS activity has been demonstrated in the livers of rats treated with VPA.^29^ As NAGS is needed to activate CPS1, the result is a decrease in carbamoyl phosphate which is the first step in the urea cycle.

Numerous case reports describe VPA‐induced hyperammonaemia in the presence of normal liver function. A 17‐year‐old female developed encephalopathy and elevated serum ammonia levels 2 weeks after the addition of VPA to her therapy with topiramate and led to an increase in serum ammonia concentrations from 94 to 252 μmol/L. Her liver function tests were normal.^30^ A 54‐year‐old male with a 14‐year history of valproate use for bipolar disorder had quetiapine added to his VPA therapy. Eight days after, he lost consciousness, had a seizure, and was found to have elevated serum ammonia with normal liver function tests.^31^ Both patients improved following the withdrawal of VPA and treatment with lactulose.

Several studies have reported hyperammonaemia with therapeutic use of VPA. Eleven of 40 patients treated with VPA for status epilepticus in an intensive care unit in Sweden developed hyperammonaemia with no correlation to the loading dose or peak serum VPA concentrations.^32^ Other studies have reported hyperammonaemia in 50% and 29/55 (53%) of patients receiving VPA.^18^, ^33^ A study of 37 patients with schizophrenia receiving VPA showed hyperammonaemia in 30% of patients, with the majority of the patients having therapeutic serum VPA concentrations. Serum ammonia concentration did not correlate with L‐carnitine concentration but there was a positive correlation between increased serum ammonia and serum glutamate concentrations.^34^

Risk factors for the development of hyperammonaemia whilst undergoing therapy with VPA include the concomitant use of other antiepileptic drugs, concomitant use of mood stabilizers, genetic variability, poor nutritional status, inborn errors in carnitine metabolism and errors in metabolism of urea, and the use of certain HIV medications.^2^

### 3.5 Valproic acid‐induced L‐carnitine deficiency

L‐carnitine concentrations can be decreased in patients with renal failure (including those undergoing haemodialysis), cirrhosis, sepsis and organ failure as a result of either a decrease in endogenous synthesis or increased loss of L‐carnitine (as in dialysis patients). VPA therapy has been implicated in causing L‐carnitine deficiency through different pathways.^35^

The first pathway includes the formation of valproylcarnitine when VPA combines with L‐carnitine. Valproylcarnitine is excreted in urine and was first discovered in the urine of children on VPA treatment in 1985.^36^ Valproylcarnitine decreases intracellular and mitochondrial levels of L‐carnitine by inhibiting the membrane carnitine transporter. Treatment with VPA has also been found to decrease the reabsorption of L‐carnitine and acylcarnitine in the renal tubule.^35^ Camina *et al*. demonstrated that mice administered VPA for 7 days had a significant increase in the renal clearance of acylcarnitine.^37^ Furthermore, VPA blocks γ‐butyrobetaine hydroxylase, an enzyme needed during the last step of L‐carnitine biosynthesis. Finally, VPA metabolism leads to a decrease in mitochondrial carnitine levels as metabolites of VPA combine with coenzyme A and carnitine palmitoyl transferase II is unable to convert acylcarnitine back into free carnitine.^35^

Meta‐analyses of studies of serum carnitine and ammonia concentrations in patients treated with VPA showed that raised ammonia was associated with reduced L‐carnitine concentrations.^34^, ^38^ Yokoyama *et al*. conducted a meta‐analysis of 50 publications and showed that blood ammonia was significantly higher and serum L‐carnitine significantly lower in patients receiving VPA.^34^

### 3.6 Acute VPA overdose

Although acute overdoses of VPA usually present as mild alteration in consciousness, large overdoses can be life‐threatening. Symptoms of toxicity include central nervous system symptoms, which can range from agitation to coma, cardiovascular toxicity (tachycardia, hypotension) and gastrointestinal toxicity (ranging from nausea and vomiting to haemorrhagic pancreatitis). Cerebral oedema, bone marrow suppression, acute kidney injury and hepatotoxicity with hyperammonaemia are more serious complications of VPA toxicity.^39^ Clinical features of toxicity occur with serum VPA concentrations greater than 450 mg/L. Coma, respiratory depression, metabolic acidosis and hypotension may occur when concentrations exceed 850 mg/L.

Treatment strategies for acute VPA overdose include supportive measures, fluid resuscitation, correction of acidosis with IV bicarbonate and, if needed, renal replacement therapy. Haemodialysis is preferred but continuous renal replacement therapy or haemoperfusion have been used. Haemodialysis can decrease the elimination half‐life of VPA to 2.5 h and help normalize metabolic parameters. Haemodialysis not only helps with the elimination of the VPA, but also lowers ammonia concentrations. The EXTRIP workgroup performed a systematic review on the use of extracorporeal treatment in VPA toxicity. VPA has some physicochemical characteristics which make it ideal for extracorporeal clearance: it has a small molecular mass, a low volume of distribution and low endogenous clearance. However, at therapeutic concentrations, it is highly protein‐bound. At serum concentrations >150 mg/L, protein binding is saturated and the free fraction of VPA increases. In acute overdoses, as concentrations of unbound VPA increase, the effectiveness of extracorporeal treatment increases. Haemodialysis and haemodiafiltration can both reach clearance rates of 90 mL/min whilst low flow continuous renal replacement techniques achieve a clearance rate of only between 10 and 15 mL/min. The EXTRIP workgroup recommends extracorporeal therapy if any of the following are present: (i) serum VPA concentrations is >1300 mg/L; (ii) cerebral oedema; or (iii) shock attributed to VPA toxicity. Extracorporeal therapy is suggested if the any of the following are present: (i) serum VPA concentration is >900 mg/L; (ii) coma or respiratory depression requiring mechanical ventilation; (iii) acute hyperammonaemia; or (iv) pH ≤ 7.10.^40^

## 4 L‐CARNITINE—SHOULD IT BE USED FOR VPA‐INDUCED TOXICITY?

### 4.1 Safety of L‐carnitine treatment

L‐carnitine is well tolerated and has a good safety profile. Apart from mild gastrointestinal upset, caution should be taken in diabetic patients as it may lower blood glucose levels.^39^ LoVecchio *et al*. reviewed the safety of L‐carnitine administration in a retrospective review of 674 poisoned patients from a single poison centre. The patients received a total of 251 doses of L‐carnitine with no adverse effects (defined as hypotension or allergic reaction in the form of wheeze or hives).^41^ Puskarich *et al*. carried out a double‐blind randomized controlled trial comparing L‐carnitine *vs*. normal saline for the treatment of septic shock. Thirty‐nine patients were enrolled in total and 16 received L‐carnitine infusion as a bolus of 4 g IV over 2–3 min, followed by a further 8 g IV over the following 12 h. There was no significant difference in safety outcome of serious adverse events between the 2 groups.^42^ A review of 8 patients with VPA toxicity who received L‐carnitine (4 IV, 2 via nasogastric tube, unknown route in 2 patients) revealed no adverse events during L‐carnitine supplementation.^43^

### 4.2 Effectiveness of L‐carnitine treatment

#### 4.2.1 L‐carnitine for chronic VPA use

There is anecdotal evidence in the form of case reports to support L‐carnitine supplementation in hyperammonaemia due to chronic VPA use (Table 2).

**TABLE 2:** L‐carnitine use in valproic acid (VPA)‐induced toxicity.

Chronic exposure to valproic acid							
Reference	Number of patients	VPA dose and/or highest concentration	Ammonia pre and post L‐carnitine	Symptoms reported	Carnitine dose	Haemodialysis	Outcome(s)
Maldonado *et al*. (2017)^44^	1	1500 mg OD 86.6 mg/L	295 μg/dL / 75 μg/dL	Seizures, headache, nausea	1 g daily	No	Not reported but symptoms presumed resolved
Cattaneo *et al*. (2017)^45^	1	Started on 1000 mg/day, increased to 1800 mg/day 73 μg/dL after 7 days, peaked at 124 μg/dL	594 μg/dL at peak. Decreased to 99 μg/dL 12 h after starting L‐carnitine	Lethargy, decreased mobility, ataxia	4.5 g/day IV	No	Discharged after resolution of symptoms. Ammonia levels decreased within 12 h of starting L‐carnitine.
Wadzinski *et al*. (2007)^46^	Case 1	1000 mg OD, 145 μg/mL	243 μmol/L / 56 μmol/L	Nonresponsive to verbal or painful stimuli	Case 1: 50 mg/kg/day	Case 1: No	Case 1: Symptoms improved/resolved.
Wadzinski *et al*. (2007)^46^	Case 2	1500 mg OD, 113 μg/mL	182 μmol/L / 80 μmol/L	Altered mental state (confusion, ataxia, slurred speech)	None	No	Symptoms improved/resolved with withdrawal of VPA, no L‐carnitine was given
Souza (2013)^47^	1	1000 mg BD, 75 mg/L	62.1 μmol/L / 7.1 μmol/L	Speech slowness, drowsiness, hemifacial clonus	1 g BD	No	Improvement at day 11, resolution in GCS/confusion

Acute VPA overdose							
Study	Number of patients	VPA dose and/or highest concentration	Ammonia levels	Symptoms reported	Carnitine dose	Haemodialysis	Outcome(s)
Chan *et al*. (2007)^48^	Case 1	20 g (400 mg/kg); peak concentration 288 mg/L at 10 h	74 μmol/L initially, max 110 μmol/L at 34 h	Drowsiness	3 g IV—received 3 doses (9 g total)	No	Asymptomatic, discharged at 48 h postingestion
Chan *et al*. (2007)^48^	Case 2	Dose ingested unknown; 950 mg/L at 1 h	43 μmol/L with maximum of 65 μmol/L	GCS 10/15, lethargy	3 g IV TDS for 3 days	No	Discharged to ward on day 5, discharged home on day 7
Ishikura *et al*. (1997)^49^	1	4 g (412.3 mg/kg); peak concentration 3289.5 μg/mL at 3 h	n/a	Deep coma, hypotonicity	100 mg/kg PO bolus, followed by 250 mg TDS for 4 days	No	Complete resolution. Consciousness level improved as VPA levels decreased.
Venkata *et al*. (2021)^50^	1	>450 μg/mL	>700 μmol/L	Coma, needed intubation	2 g IV bolus, followed by 1.625 g every 4 h	Yes: CVVHD	Extubated on day 3, discharged on day 9
Pagali *et al*. (2021)^7^	1	3 g ingested 49 mg/kg; Free VPA 134 μmol/L (normal 5–25 μmol/L)	70 μmol/L	Headache, blurred vision, agitation	Initial dose of 6 g IV (100 mg/kg), further 15 mg/kg 8 hourly, further doses at 50 mg/kg 8 hourly	Yes: haemodialysis needed on 2 occasions during same admission	Full recovery and discharge.

Abbreviations: BD, twice daily; GCS, Glasgow Coma Scale; IV, intravenous; OD, once daily; PO, oral; TDS, 3 times/day.

One study found that supplementation with L‐carnitine (50 mg/kg/day) for 4 weeks corrected both L‐carnitine concentrations and hyperammonaemia in a group of 14 patients receiving VPA.^18^

Okumura *et al*. reported on the effects of L‐carnitine supplementation in 69 paediatric patients treated with VPA for childhood epilepsy. They measured serum free carnitine and acylcarnitine levels, VPA concentrations, liver function tests, serum amylase levels and serum ammonia levels. Eight out of 69 patients were prescribed L‐carnitine (between 250 mg and 1000 mg/day) due to carnitine deficiency or being on a ketogenic diet. The patients receiving L‐carnitine supplementation had higher serum free carnitine levels. Serum free carnitine levels were found to be decreased in 32 out of 61 patients receiving VPA but not in those with L‐carnitine supplementation. Whilst the authors noted a mild elevation in ammonia levels in patients not receiving L‐carnitine supplementation, this was not statistically significant.^51^

Nakamura *et al*. prospectively supplemented 22 psychiatric patients receiving VPA for 3 or more months and had elevated serum ammonia levels (>86 μg/dL). A fixed dose of L‐carnitine (30 mg/kg/day) was administered for 3 months with serial measurements of serum ammonia, VPA, free, acyl‐ and total carnitine, and ratios of free to acylcarnitine. Supplementation with oral L‐carnitine resulted in an increase in free, acyl‐ and total carnitine levels. About half of the patients had reduced serum ammonia after L‐carnitine supplementation (ammonia concentration at start of therapy 109.4 ± 5.6 μg/dL *vs*. 83.5 ± 6.2 μg/dL at 3 months of supplementation).^52^

Bohan *et al*. carried out a retrospective inception cohort study to determine whether L‐carnitine therapy resulted in better hepatic survival amongst patients who experienced acute, severe, symptomatic valproate‐induced hepatotoxicity. The primary outcome was hepatic survival (deceased and liver transplant patients were classified as hepatic failure). Hepatic survival occurred in 47% of the 92 patients who received L‐carnitine (orally or IV) compared to 10% of the 50 historical controls who did not receive L‐carnitine. Intravenous administration was associated with an increased hepatic survival (14/21 *vs*. 6/21), particularly when administered within the first 5 days.^53^

Glatstein *et al*. retrospectively analysed the charts of 13 patients (all under the age of 16) who presented with VPA‐induced hyperammonemia and encephalopathy between 2012 and 2015. Of the 13 patients, 11 were on long‐term VPA therapy and 2 presented after an acute overdose. All patients received IV L‐carnitine. Twelve patients received a daily dose of 100 mg/kg and 1 patient received a daily dose of 200 mg/kg. None of the patients received extracorporeal therapy. The most severe case, which presented as an acute overdose, had a peak serum valproate of 582 μg/mL and a serum ammonia of 138 μmol/L. The patient received IV L‐carnitine and their encephalopathy resolved after 2 days without the need for haemodialysis. Time to normalization of serum ammonia in this study ranged from 13 to 60 h, whilst other studies have reported times ranging from 7 to 70 h. None of the patients encountered any adverse effects.^54^

#### 4.2.2 L‐carnitine for acute VPA overdose

The effectiveness of L‐carnitine for acute VPA overdose is less clear. The benefit of L‐carnitine as an antidote in VPA overdose remains a controversial subject and based largely on case reports. As a result of its good safety profile and low cost, L‐carnitine is often recommended in individuals who have taken an overdose if the serum concentration is 450 mg/L or higher and they present with coma, hyperammonaemia or hepatotoxicity.

Murakami *et al*. describe the case of a 15‐month‐old child who ingested 4 g of VPA and presented in deep coma. There was an increase in the 4‐en‐VPA metabolite of VPA. Administration of L‐carnitine resulted in a restoration of normal VPA metabolism. The child recovered on day 3 of therapy without the need for haemodialysis.^55^ Other case reports are summarized in Table 2. While these case reports suggest a role for L‐carnitine therapy, the concomitant use of haemodialysis in several of the cases makes it difficult to establish whether the decrease in serum ammonia resulted from haemodialysis or L‐carnitine or both. In some case reports, haemodialysis was not used and appeared to provide anecdotal evidence that IV L‐carnitine use can alter VPA metabolism after acute massive overdoses and alter patient outcomes.^48^, ^49^

In a randomized controlled study of 62 patients with acute valproate overdose and valproic concentrations >150 mg/L, 34 patients received standard treatment and 28 received 1800 mg/day L‐carnitine for 3 days in addition to standard treatment. L‐carnitine supplementation resulted in a statistically significant reduction in ammonia after 24 h (47.9 ± 6 μmol/L) compared to standard treatment (61.9 ± 11.39 μmol/L).^56^

Nguyen *et al*. studied 69 patients admitted to intensive care with VPA toxicity and calculated predicted *vs*. observed VPA half‐lives in patients who received L‐carnitine or did not, along with changes to serum lactate levels and changes in scores Sequential Organ Failure (SOFA) score. 19 out of the 69 patients received IV L‐carnitine (100 mg/kg loading dose followed by 3 g/day in 3 equally divided doses for 3 days or until discharge from intensive care unit [ICU]) and 2 patients required haemodialysis. The main biochemical findings in the 69 patients were elevated serum lactate levels (mean 2.9 mmol/L) and elevated serum ammonia levels (mean 96 μmol/L). Two patients who developed hyperammonaemia and cerebral oedema appeared to improve after administration of L‐carnitine. However, L‐carnitine administration did not alter VPA elimination or change in SOFA scores but may have an effect on the rate of serum lactate normalization. However, patients treated with L‐carnitine had higher peak serum ammonia levels (227 *v*s. 124 mmol/L), higher peak serum VPA concentrations (287 *vs*. 231 mg/L) and peak serum lactate levels (4.6 *vs*. 3.5 mmol/L). Mean length of stay on ICU was higher in those treated with L‐carnitine (5 days) compared to those not treated with L‐carnitine (2 days) suggesting that the cohort treated with L‐carnitine was more severely intoxicated.^19^

It is difficult to establish whether L‐carnitine therapy improves mortality in patients with VPA toxicity. An observational retrospective study described favourable outcomes in 316 patients who presented to hospital with VPA toxicity and did not receive L‐carnitine therapy as it is not readily available in Iran. All patients received standard care including IV fluids, activated charcoal, gastrointestinal lavage and admission to ICU for organ support or haemodialysis if needed; 14 patients required intubation, 3 required dialysis and 2 patients died. The limitation of this case series is the lack of complete data on peak serum VPA concentrations.^57^

#### 4.2.3 Dosing of L‐carnitine for VPA‐induced toxicity

Schiavo *et al*. developed a quantitative systems pharmacology model to evaluate VPA‐induced hyperammonaemia and used this model to evaluate the use of L‐carnitine in preventing hyperammonaemia in both chronic and acute VPA toxicity. The model was able to predict total and unbound VPA plasma concentrations, along with concentrations of VPA metabolites (VPA‐glucuronide, 2‐en‐VPA and 4‐en‐VPA) for both single and multiple dosing. The model suggested that the dose of L‐carnitine required to prevent hyperammonaemia ought to be double the VPA dose, for instance, an individual taking 500 mg VPA daily would require 1000 mg L‐carnitine orally to maintain normal serum ammonia. The model was also used to predict the impact of IV L‐carnitine as rescue therapy in acute overdoses (5 g) of VPA in individuals already established on chronic VPA therapy (500 mg twice daily). Intravenous administration of 6 g of L‐carnitine 2 h after overdose, followed by six 1 g IV boluses every 4 h, was predicted to reduce serum ammonia. The model predicted hyperammonaemia persisting for approximately 60 h if IV L‐carnitine was not administered. Delayed administration of IV L‐carnitine up to 8 h post‐overdose was found to have a positive effect in reducing duration of hyperammonaemia.^58^

In the UK, the current dosing recommendations for IV L‐carnitine therapy in patients displaying toxicity following chronic or acute exposure is a bolus of 100 mg/kg IV over 30–60 min (maximum of 6 g), followed by a maintenance dose of 15 mg/kg IV every 4 h.^39^

However, these dosing guidelines are empirical and based on case reports and do not take into account the pharmacokinetics of exogenously administered L‐carnitine. The aim of L‐carnitine administration is to achieve serum L‐carnitine concentrations which enable redistribution to the liver where VPA causes a relative depletion of L‐carnitine. The poor oral bioavailability of L‐carnitine makes the oral route unsuitable. Intravenous boluses of L‐carnitine lead to rapid renal excretion due to saturated renal reabsorption limiting the redistribution from plasma to liver. Assuming physiological L‐carnitine concentrations around 40–60 μmol/L, targeting a therapeutic L‐carnitine concentration of 100–150 μmol/L (16–24 mg/L) appears reasonable. Based on the reported initial volume of distribution of 0.2–0.3 L/kg after IV dosing, an IV bolus dose of 30 mmol/kg (5 mg/kg) L‐carnitine is sufficient to achieve a concentration of 100–150 μmol/L. Assuming renal clearance of L‐carnitine following IV dosing is 75–100 mL/min, a low‐dose continuous infusion of 1–2 mg/kg/h is expected to maintain L‐carnitine concentrations of 100–150 μmol/L. In patients receiving haemodialysis, it is unclear whether L‐carnitine should be continued given that haemodialysis is very effective at clearing ammonia. If L‐carnitine is continued, the infusion rate should be increased to 5–10 mg/kg/h during haemodialysis.

## 5 DISCUSSION

VPA is commonly used for epilepsy, bipolar disorder and migraine prophylaxis. Whilst most patients tolerate the drug well, a proportion develop side‐effects ranging from a transient elevation of liver enzymes to fulminant liver failure. The metabolism of VPA can be affected by numerous factors including genetic variability in CYP450 enzymes, inborn errors of metabolism, concomitant use of other medications, malnutrition and inhibition of normal metabolic pathways during acute poisoning. Hyperammonaemia due to VPA use, both in chronic use and after overdose, is a recognized phenomenon and is the result of altered VPA metabolism.

Serum L‐carnitine appears to be lower in individuals taking VPA long‐term. Given that serum L‐carnitine represents a very small proportion of total body carnitine, it is unclear whether this is the most appropriate measure of total body stores in acute overdose. In chronic VPA use, it is probably a more reliable indicator of total body stores. There are numerous case reports demonstrating reduction in serum ammonia following use of L‐carnitine in both chronic VPA therapy and after acute poisoning. However, a recent study found no clinical benefit of IV L‐carnitine in enhancing VPA elimination or improving organ dysfunction following acute overdose.

Oral L‐carnitine may be considered for long‐term supplementation in patients receiving VPA therapy to prevent hyperammonaemia. However, in acute VPA overdose, IV L‐carnitine is preferred but the most appropriate dose and duration of treatment remains unclear. The doses used in case reports involved IV boluses, which are likely to give rise to a large proportion of the administered dose being renally excreted due to saturation of renal tubular reabsorption. Based on pharmacokinetic considerations, it is proposed that an IV bolus dose of 5–10 mg/kg followed by a continuous infusion of 1–2 mg/kg/h is more likely to achieve sustained supraphysiological concentrations of L‐carnitine. However, this theoretical dose has not been implemented in clinical practice and requires further research.

Given the good safety profile of L‐carnitine, it is reasonable to use L‐carnitine in patients with acute valproate overdose associated with hyperammonaemia or hepatotoxicity. However, given the paucity of evidence that it alters clinical outcomes, other modalities of treatment including enhancing elimination of VPA with carbapenems and/or extracorporeal treatment should be considered concomitantly.

## AUTHOR CONTRIBUTIONS

Tomasz Gziut and Ruben Thanacoody independently reviewed the literature and both contributed to drafting the manuscript.

## CONFLICT OF INTEREST STATEMENT

The authors have no conflict of interest to declare.

## Data Availability

Data sharing is not applicable to this article as no new data were created or analyzed in this study.
